# Co-occurrence of antibiotic and metal resistance in long-term sewage sludge-amended soils: influence of application rates and pedo-climatic conditions

**DOI:** 10.1007/s11356-022-23802-2

**Published:** 2022-11-12

**Authors:** Sonia Mokni-Tlili, Sarra Hechmi, Hadda-Imene Ouzari, Najet Mechergui, Manel Ghorbel, Naceur Jedidi, Abdennaceur Hassen, Helmi Hamdi

**Affiliations:** 1 Water Research and Technology Center, University of Carthage, P.O. Box 273, 8020 Soliman, Tunisia; 2 Laboratory of Microorganisms and Active Biomolecules, Faculty of Sciences of Tunis, University of Tunis El Manar, LR03ES03 Tunis, Tunisia; 3 Food and Water Security Program, Center for Sustainable Development, College of Arts and Sciences, Qatar University, P.O. Box 2713, Doha, Qatar

**Keywords:** Urban sewage sludge, Organic amendment, Agricultural soils, Antibiotic-resistant bacteria, Metal resistance, Flash flood

## Abstract

Urban sewage sludge (USS) is increasingly being used as an alternative organic amendment in agriculture. Because USS originates mostly from human excreta, partially metabolized pharmaceuticals have also been considered in risk assessment studies after reuse. In this regard, we investigated the cumulative effect of five annual USS applications on the spread of antibiotic-resistant bacteria (ARB) and their subsequent resistance to toxic metals in two unvegetated soils. Eventually, USS contained bacterial strains resistant to all addressed antibiotics with indices of resistance varying between 0.25 for gentamicin to 38% for ampicillin and azithromycin. Sludge-amended soils showed also the emergence of resistome for all tested antibiotics compared to non-treated controls. In this regard, the increase of sludge dose generally correlated with ARB counts, while soil texture had no influence. On the other hand, the multi-antibiotic resistance (MAR) of 52 isolates selected from USS and different soil treatments was investigated for 10 most prescribed antibiotics. Nine isolates showed significant MAR index (≥ 0.3) and co-resistance to Cd, As and Be as well. However, events including an extreme flash flood and the termination of USS applications significantly disrupted ARB communities in all soil treatments. In any case, this study highlighted the risks of ARB spread in sludge-amended soils and a greater concern with the recent exacerbation of antibiotic overuse following COVID-19 outbreak.

## Introduction

Antibiotics are essential medical drugs that have increasingly been used to treat the majority of bacterial infections in humans and animals. According to a recent report published by the World Health Organization on antibiotic consumption in 65 countries (WHO 2018), Defined Daily Doses (DDD) per 1000 inhabitants per day varied between 4.4 and 64.4, and the overall absolute quantity varied from 1.3 to 2225 tonnes per year per country. On the other hand, these molecules are partially metabolized in human and animal bodies after administration, resulting in their presence in relevant amounts in excreta (Kümmerer and Henninger 2003; Looft et al. 2012; Michael et al. 2013; Zhou et al. 2021). Consequently, the excessive use of antibiotics during recent years has led to serious problems due to their environmental release and the spread of antibiotic-resistant bacteria (ARB) (Ronquillo 2017; Vikesland et al. 2017; Cycon et al. 2019). This overuse has even been worsened by the COVID-19 pandemic particularly during the first stages of emergency clinical management (Yacouba et al. 2021). Currently, several hundred thousands of deaths every year could be attributed to antibiotic-resistant bacteria (Cassini et al. 2019; Gasser et al. 2019; Raoult et al.^ 2019^). According to the Proceedings of the National Academy of Sciences of the United States of America, Tunisia was classified second behind Turkey in terms of worldwide antibiotics consumption between 2000 and 2015 (Klein et al. 2018). Moreover, several antibiotics of human usage are resistant to different conventional wastewater treatment steps as well and end up partitioned between treated effluents and the suspended particles (Golet et al. 2002; Osińska et al. 2019).

Urban sewage sludge (USS) is the semi-solid by-product of wastewater treatment process resulting from the settling of suspended solids. It has a complex composition that includes organic carbon, macro- and micronutrients and various classes of pollutants (Hamdi et al. 2007). Besides the chronic issue of contamination with heavy metals and soluble salts, various organic pollutants and emerging compounds including antibiotic residues have consistently been found in urban sludge (Osińska et al. 2019; Hechmi et al. 2020). In addition to routine investigations on the well-studied effects of sludge-borne metals (Elmi et al. 2020), other “stressors” may affect the survival of various microbial communities because different organisms have different degrees of tolerance or defensive responses (Seiler and Berendonk 2012; Berendonk et al. 2015; Manaia et al. 2016). Moreover, the presence of contaminants, together with nutrients, stable pH and temperature, and the close proximity of cells in the stabilized sewage sludge might on one hand create a natural environment for the selection of adapted microorganisms and on the other hand stimulate the acquisition of resistance against these stressors (Buta et al. 2021). For instance, residual antibiotics in USS facilitate the spread of ARB through horizontal transfer of resistance-encoding genes (Dröge et al. 2000; Kim et al. 2014; Di Cesare et al. 2016; Marano and Cytryn 2017).

Because USS has increasingly been used as an alternative organic fertilizer in agriculture (Hamdi et al. 2019; Hechmi et al. 2020; Markowics et al. 2021), it represents along with livestock manure the major sources for the transfer of residual antibiotics and antibiotic-resistance genes (ARGs) to croplands (Munir and Xagoraraki 2011; Burch et al. 2014; Bondarczuk et al. 2016; Singer et al. 2016; Zhang et al. 2019). Consequently, the increasing resistome in amended soils can potentially enter the food chain via contaminated crops, representing an important vehicle for ARGs transmission into human microbiome (Chen et al. 2019a, b; Zhang et al. 2019). This issue could be of particular concern in agricultural countries like Tunisia or Turkey where antibiotic consumption and sludge reuse have been increasing concurrently (Klein et al. 2015; Mansour 2018; Hechmi et al. 2020). Nevertheless, data regarding the extent of antibiotic resistance in agricultural soils due to sludge application remain limited globally and inexistent locally (Bondarczuk et al. 2016; Markowics et al. 2021). Therefore, the main objective of this agro-environmental study was to determine antibiotic-resistant bacteria and their concomitant resistance to toxic metals in USS and long-term sludge-amended soils. In this regard, the influence of sludge dose, repetitive applications, soil texture and an unexpected climatic hazard were all studied under field semi-arid conditions.

## Materials and methods

### Site description and experimental protocols

Field trials using urban sewage sludge as an organic amendment were carried out in an agricultural experiment station located in northeastern Tunisia (36° 27′ 15″ N, 10° 44′ 5″ E). The region is characterized by typical south Mediterranean light-textured soils and a superior semi-arid climate with moderate but irregular precipitation (350–400 mm per year). Two close experimental plots of 400 m^2^ each were chosen according to soil texture, namely, a sandy soil (S) and a sandy loam soil (SL). Both soils are predominantly coarse, but soil SL has higher clay fraction, specific surface area (SSA), cation-exchange capacity (CEC), total organic carbon (TOC) and soil organic matter (SOM) contents (Table 1). Moreover, XRD analysis of soil SL revealed the presence of illite and kaolinite as the main clay minerals (Hamdi et al. 2019). The aerobically digested sludge used in this study was collected from the drying beds of a close urban wastewater treatment plant. As for most urban sludges, it has not only significantly higher TOC and N contents than both soils (22.5% and 1.5%, respectively) but also substantial salinity and heavy metal concentrations (Table 1).

**Table 1:** Physico-chemical properties of experimental soils and urban sewage sludge

Parameters*	Sandy soil (S)	Sandy loam soil (SL)	Urban sewage sludge (USS)
Sand (%)	83.3	70.9	-
Silt (%)	11.5	17.2	-
Clay (%)	5.2	11.9	-
SSA (m^2^ g^−1^)	13	27	-
CEC (meq 100 g^−1^)	1.8	2.2	-
pH	7.24	7.72	7.6
EC (µS cm^−1^)	119	155	3100
Na (mg kg^−1^)	80	196	1840
K (mg kg^−1^)	84.4	58.8	119
Ca (g kg^−1^)	1.54	9.56	121.6
CaCO_3_ (%)	0.91	1.76	11.8
TOC (%)	0.67	0.76	22.5
SOM (%)	1.15	1.30	-
Total N (%)	0.1	0.071	1.5
C:N	6.7	10.7	15
P Olsen (mg kg^−1^)	17.5	14.1	235
Total Ni (mg kg^−1^)	0.58	0.44	23
Total Zn (mg kg^−1^)	5.88	2.48	320
Total Cu (mg kg^−1^)	1.37	0.09	125
Total Pb (mg kg^−1^)	16.2	16.5	30
Total Cr (mg kg^−1^)	0.69	0.65	67
Total Cd (mg kg^−1^)	0.36	0.74	4.04
Total As (mg kg^−1^)	0.44	0.62	17
Total Be (mg kg^−1^)	10	11.1	10
Bacteria (× 10^5^ CFU g^−1^)	103	122	870

^*^All parameters are given on dry weight basis

The field study consisted of repeated annual applications of USS to both soils performed for 5 consecutive years starting from 2012. More precisely, USS had been added annually to 4-m^2^ elementary plots at equivalent field application rates of 40, 80 and 120 tons of dry sludge weight ha^−1^ year^−1^ to cover a wide range of USS doses. Control was unamended soil, and all treatments were conducted in quadruplicate in a randomized complete block design. Treatments were named according to soil type and USS dose as follows: S0 (control), S40, S80 and S120 for soil S and SL0 (control), SL40, SL80 and SL120 for soil SL. For each annual amendment, USS was incorporated in the topsoil layer during autumn (October–November), which corresponds to the beginning of the cool rainy season and agricultural activities in the region. Throughout the investigation period, all elementary plots were kept consistently bare by manual weeding to avoid the effect of herbicide use and/or plant presence on soil properties. Consequently, changes in soil physico-chemical properties and microbiome were exclusively influenced by the sludge dose and prevailing pedo-climatic conditions.

Routine topsoil samplings had been performed each year prior to the subsequent sludge application. Soil samples described in this study were collected after five annual USS amendments (fall 2017) at 0–20 cm depth, put into sterilized bags and promptly stored at − 80 °C prior to analysis. The physico-chemical properties of these samples are available in Hechmi et al. (2020). This was supposed to be the fifth and final planned sampling campaign for this long-term field study. However, the experimental site experienced a devastating flash flood on September 22, 2018 (almost 1 year after the last scheduled soil sampling) with about 200 mm of rain fallen in just few hours. Consequently, an improvised sampling campaign was carried out when the field had become accessible to investigate the effect of such extreme climatic event on soil microbial properties as compared to those observed during the last regular sampling campaign.

### Total and antibiotic-resistant bacteria

Culture-based approaches were employed for bacterial enumeration in USS and all soil samples taken during both sampling campaigns, namely, (i) after five successive sewage sludge amendments (fall 2017) and (ii) after the flash flood of the following year (fall 2018). Total heterotrophic bacteria (THB) and bacteria resistant to each of the following antibiotics, namely, amoxicillin (AMX), ampicillin (AMP), azithromycin (AZI), doxycycline (DOX), erythromycin (ERY), gentamicin (GEN) and levofloxacin (LEV), were determined in Reasoner’s 2A (R2A) agar (Udikovic-Kolic et al. 2014; Zhang et al. 2018). These antibiotics are the most prescribed in Tunisia according to the Company of Pharmaceutical Industries of Tunisia (SIPHAT) and an internal survey performed by the research team among local pharmacists and doctors. Antibiotic concentrations in culture media were chosen according to the European Committee on Antimicrobial Susceptibility Testing (EUCAST) as follows (Matuschek et al. 2014): AMX (32 mg L^−1^), AMP (32 mg L^−1^), DOX (16 mg L^−1^), AZI (4 mg L^−1^), ERY (8 mg L^−1^), LEV (2 mg L^−1^) and GEN (4 mg L^−1^). Before use, antibiotic solutions were sterilized by filtration through 0.22-µm pore size. Cycloheximide was added to the R2A agar medium at 100 mg L^−1^ to inhibit fungal development.

To isolate microorganisms from sludge and soil, fresh samples were first stirred in sterilized phosphate-buffered saline (PBS) at a ratio of 10 g to 90 mL for 2 h. The supernatant was then serially diluted to the appropriate concentrations with PBS. Aliquots (100 μL) of each dilution were spread onto corresponding culture plates with negative controls consisting of 100 μL of PBS spread onto similar plates. The number of colony-forming units (CFU) was determined after 5 days of incubation at 28 °C using a colony counter (Udikovic-Kolic et al. 2014). All assays were performed in triplicate. The index of antibiotic resistance (IAR) was determined as the ratio between the number of colony-forming units (CFU) in presence of each antibiotic (ARB) and the total heterotrophic bacteria (THB) of the same sample as follows:

$$\%IAR = (ARB/THB)\times 100$$

At the end of this first investigation, we proceeded to the isolation of representative colonies from each morphological group observed on THB and ARB plates of USS and all soil samples. It resulted in 52 isolates that were purified and used for the subsequent investigation on multiple antibiotic and heavy metal resistance.

### Multiple antibiotic resistance test

The disc diffusion test was used to examine the multiple resistance of the isolated colonies against 10 antibiotics, namely, penicillin (PEN), ofloxacin (OFL), chloramphenicol (CHL), AMX, AMP, DOX, ERY, AZI, LEV and GEN. Among these, three are classified as β-lactams (PEN, AMX and AMP), two macrolides (ERY and AZI), two fluoroquinolones (OFL, LEV), one tetracycline (DOX), one aminoglycoside (GEN) and one phenicol (CHL). A volume of 20 μL of each antibiotic solution was deposited on sterile Whatman No. 17 discs (Ø 6 mm) before placement on a lawn of isolated bacteria grown on Mueller–Hinton agar. Plates were examined after incubation of 24 h at 30 °C. When existing, clear zones of inhibition (halos) around discs were measured and compared to the CLSI standard values for significance (CLSI 2012). An index of multiple antibiotic resistance (MAR) could be then calculated as the ratio between the number of antibiotics to which an isolate is resistant and the total number of tested antibiotics (Kimiran-Erdem et al. 2015; Jardine et al. 2019). Multiple resistance of a given bacterial strain is considered when confirmed for at least three antibiotics (Jardine et al. 2019). Therefore, multiple antibiotic resistance of an isolate was considered significant in the current study when MAR index ≥ 0.3.

### Heavy metal resistance assay

In addition to antibiotic resistance assessment, a separate batch of culture media was prepared to investigate metal-resistant bacteria (MRB). The aim of this assay was to determine the percentage of bacteria having dual metal and antibiotic resistance capacities, therefore, the co-occurrence of these two properties. The resistance of the 52 isolated colonies to heavy metals was determined by disc diffusion assay as well (Jardine et al. 2019). Three soil- and USS-borne toxic metals were selected for this test, namely, cadmium (Cd), arsenic (As) and beryllium (Be) (Table 1). The specific choice of Cd and As was based on their potential toxic effects to agricultural plants and humans (Clemens and Ma 2016; Balali-Mood et al. 2021). Besides, beryllium was found in USS and both experimental soils despite the fact that its environmental occurrence and biological toxicity have rarely been addressed in routine studies on sludge/soil contamination. As for antibiotic susceptibility testing, each solution of trace metal was applied on a disc deposed on isolated bacterial lawns grown on a Mueller–Hinton medium. The concentration of each trace metal was set at 20 mg L^−1^, an extremely high content for bioavailable (dissolved) toxic metals in the pore water of both soils (Hechmi et al. 2020). Inoculated plates were incubated at 37 °C for 24 h, and then inhibition zones surrounding metal-soaked discs were monitored. The strain was considered resistant to a given metal when inhibition zones around discs were 1 mm or less (Tomova et al. 2015).

### Bacteria identification using 16S rDNA sequencing

A selected isolate from each bacterial group that presents a resistance against the same antibiotic(s) and/or metal(s) was purified and prepared for molecular identification. The extraction of DNA was carried out using the FastDNA® Spin kit for bacteria (6560–200). PCR amplification was performed targeting 16S rDNA genes using F27 (5′-AGAGTTTGATCMTGGCTCAG-3′) and R1492 (5′-TACGGYTACCTTGTTACGACTT-3′) primers (Monciardini et al. 2002). Amplification reactions were performed in a final volume of 25 µL, containing 50 mM KCl, 10 mM Tris–HCl (pH 8.3), 2.0 mM MgCl_2_, 0.2 mM dNTP, 1.0 μM of each primer and 2.5 U Taq polymerase. The PCR cycle was set as follows: initial denaturation at 94* °C* for 5 min followed by 35 cycles of denaturation at 94 °C for 1 min, annealing at 55 °C for 1 min and extension at 72 °C for 2 min, with a final extension at 72 °C for 7 min. Amplified products were visualized on ethidium bromide stained agarose gel (1% w/v). Positive extracts were purified by the QIAquick PCR Purification Kit (Qiagen) and sequenced using ABI PrismTM 3100 (Perkin Elmer Applied Biosystems). The obtained sequences were deposited in GenBank under accession numbers from MT279584 to MT279592.

### Phylogenetic assignment and statistical analysis

The effect of USS dose on THB and ARB variation in each soil type was analysed by ANOVA with a post hoc Duncan’s multiple range test (*P* ≤ 0.05) for quadruplicate mean separation at each sampling date (STATISTICA 8.0, StatSoft Inc., Tulsa, USA). Pearson product–moment correlation coefficients (*r*) estimated the strength of relationship at *P* ≤ 0.05. Obtained sequences were aligned with sequences within the GenBank database by BLAST. A phylogenetic tree was constructed using the online software NGPhylogeny (https://ngphylogeny.fr). Phylogenetic relationships between tested sequences and similar sequences retrieved by BLAST were inferred based on 100 bootstrap replications and the neighbour joining method.

## Results

Outcomes are principally reported and discussed for soil samples collected in 2017, which describe the effect of five annual USS applications with increasing rates under normal climate conditions. Those of 2018 highlight the consequences of an extreme autumnal flash flood on counts and distribution of total and antibiotic-resistant bacteria for the same soil treatments.

### Total and antibiotic-resistant bacteria

Results of cultivable THB and ARB are presented in Table 2. The used urban sewage sludge showed the highest number of THB (870 × 10^5^ CFU g^−1^) compared to all soil treatments, while untreated controls had the lowest bacterial counts (137 × 10^5^ and 104 × 10^5^ CFU g^−1^ for S0 and SL0, respectively). Sewage sludge addition positively affected THB populations in both soils in a significant dose-dependent manner as compared to control treatments (Table 2). Therefore, highest THB counts in USS-amended soils were observed for treatments S120 and SL120 (302 × 10^5^ and 608 × 10^5^ CFU g^−1^, respectively). It is worth mentioning that the numbers of heterotrophic bacteria in soil SL were consistently higher than in soil S when same treatments are compared two by two (e.g. 152 × 10^5^ and 453 × 10^5^ CFU g^−1^ for S40 and SL40, respectively).

**Table 2:** Total heterotrophic bacteria (THB) and antibiotic-resistant bacteria (ARB) in urban sewage sludge (USS) and different soil treatments

Sample	CFU (× 10^5^ g^−1^ dry soil)														
	*THB*	*ARB*													
		*AMX*	%IAR*	*AMP*	*%IAR*	*DOX*	*%IAR*	*ERY*	*%IAR*	*AZI*	*%IAR*	*LEV*	*%IAR*	*GEN*	*%IAR*
USS	870 ± 212	153 ± 17	*17.6*	332 ± 42	*38.2*	208 ± 27	*23.9*	88 ± 7.5	*10.1*	332 ± 24	*38.2*	150 ± 43	*17.2*	2.25 ± 0.65	*0.25*
Sampling of fall 2017**															
S0	137 ± 16 a	5.2 ± 0.8 a	*3.7*	nd	-	nd	-	5.05 ± 0.86 a	*3.7*	2.95 ± 1.1 a	*2.1*	0.9 ± 0.4 a	*0.7*	nd	-
S40	152 ± 27 ab	72 ± 11 b	*47.4*	8 ± 0.7 a	*5.3*	nd	-	89 ± 13 c	*58.5*	48.5 ± 12 b	*31.9*	12 ± 3.1 b	*7.9*	nd	-
S80	163 ± 15 b	137 ± 72 c	*84*	95 ± 12 b	*58.3*	nd	-	26 ± 7.1 b	*15.9*	124 ± 31 c	*76.1*	nd	-	nd	-
S120	302 ± 23 c	250 ± 38 d	*82.8*	183 ± 16 c	*60.8*	nd	-	100 ± 9 c	*33.1*	200 ± 23 d	*66.2*	nd	-	nd	-
*Corr*	***0.85***	***0.99***	*-*	***0.95***	*-*	-	-	***0.61***	*-*	***0.99***	*-*	− 0.32	-	-	-
SL0	104 ± 12 a	nd	-	nd	*-*	nd	-	2.5 ± 0.16 a	*2.4*	1.12 ± 0.04 a	*1.1*	nd	-	nd	-
SL40	453 ± 17 b	149 ± 62 b	*33.9*	136 ± 44 ab	*30*	120 ± 12 a	*26.5*	25 ± 7.7 b	*5.5*	31 ± 3.5 b	*6.8*	nd	-	nd	-
SL80	455 ± 23 b	104 ± 43 a	*22.8*	94 ± 24 a	*20.6*	163 ± 11 a	*35.8*	50 ± 12 c	*11*	50 ± 12 c	*11*	32 ± 2.5 b	*7*	17 ± 1.3 b	*3.7*
SL120	608 ± 71 c	191 ± 57 c	*31.4*	272 ± 39 b	*44.7*	nd	-	90 ± 8.4 d	*14.8*	92 ± 19 d	*15.1*	3.5 ± 0.17 a	*0.6*	3.5 ± 0.02 a	*0.6*
*Corr*	***0.92***	***0.83***	*-*	***0.88***	*-*	0.07	-	***0.99***	*-*	***0.99***	*-*	0.35	*-*	0.44	*-*
Sampling of fall 2018***															
S0	13 ± 7 a	nd	-	nd	-	nd	-	nd	*0*	0.124 ± 0.1 a	*0.96*	nd	-	nd	-
S40	40 ± 26 b	nd	-	nd	-	nd	-	3 ± 1.8 a	*7.5*	1 ± 0.05 a	*2.5*	nd	-	nd	-
S80	52 ± 18 c	nd	-	nd	-	nd	-	11 ± 3.7 b	*21.1*	10 ± 5.4 b	*19.2*	nd	-	nd	-
S120	57 ± 25 c	nd	-	nd	-	nd	-	24.5 ± 12 c	*43*	19 ± 5.6 c	*33.3*	nd	-	7 ± 2.3	*12.3*
*Corr*	***0.94***	-	-	-	-	-	-	***0.94***	*-*	***0.96***	*-*	-	-	0.37	*-*
SL0	33 ± 13 a	nd	-	nd	-	nd	-	2 ± 0.91 a	*6.1*	28 ± 5.5 b	*84.9*	nd	-	nd	-
SL40	105 ± 41 b	5 ± 2.2 a	*4.8*	1.25 ± 0.3 a	*1.2*	nd	-	15.5 ± 6 b	*14.8*	72.5 ± 14 d	*69*	4 ± 1.6 a	*3.8*	nd	-
SL80	297 ± 67 c	102 ± 56 c	*34.3*	91 ± 11 c	*30.7*	1	*0.3*	28 ± 11 c	*9.4*	50 ± 21 c	*16.8*	7 ± 2.1 a	*2.3*	2 ± 0.75	*0.6*
SL120	358 ± 102 d	25 ± 12 b	*7*	46 ± 6.5 b	*12.8*	nd	-	100 ± 44 d	*27.9*	4 ± 1.4 a	*1.1*	23 ± 12 b	*6.4*	nd	-
*Corr*	***0.98***	0.47	*-*	***0.68***	*-*	0.26	-	***0.90***	*-*	− 0.41	*-*	***0.92***	*-*	0.26	-

^*^%IAR: index of antibiotic resistance (ARB/THB)

^**^After five annual USS applications under normal conditions

^***^After a devastating flash flood

*nd* Not detected

For each soil type (S or SL) and each microbial parameter, means with same letters are not statistically different at *P* ≤ 0.05. Bold italic Pearson product–moment correlation coefficients are significant at *P* ≤ 0.05

The urban sewage sludge contained cultivable bacterial strains resistant to all studied antibiotics with IAR values varying between 0.25 for GEN to 38.2% for AMP and AZI (Table 2). Significant bacterial populations resistant to DOX (208 × 10^5^ CFU g^−1^), AMX (153 × 10^5^ CFU g^−1^) and LEV (150 × 10^5^ CFU g^−1^) were also observed in the sludge sample. On the other hand, both untreated soils S0 and SL0 did not originally contain any bacterial strains resistant to AMP, DOX and GEN but shared resistance to AZI (2.1% and 1.1%, respectively) and ERY (3.7% and 2.4%, respectively). However, AMX- and LEV-resistant bacteria existed only in S0 treatment. Five successive annual applications of sludge resulted in the appearance of resistant bacteria to all tested antibiotics in both USS-treated soils though at a large variability with treatments. In general, resistant bacteria to ERY, AZI, AMX and to a lesser extent AMP were the most abundant in all soil treatments (Table 2). In particular, soils treated with USS at 120 t ha^−1^ year^−1^ showed consistently the highest ARB counts with highest numbers observed for AMX (250 × 10^3^ CFU g^−1^) and AMP (272 × 10^3^ CFU g^−1^) in S120 and SL120, respectively. In contrast, the lowest ARB counts were generally observed in untreated controls (S0 and SL0). Accordingly, there was a general trend of positive correlations between USS dose and ARB counts in both soils in spite of large variations between antibiotic types (Table 2). In this regard, AZI showed the strongest correlation with USS dose in both soils (*r* = 0.99), while LEV-resistant bacteria counts were not seemingly affected by sludge application rates. Besides, it is worth mentioning that the textural effect on ARB distribution between both soils was not as obvious as with total heterotrophs (Table 2).

On the other hand, bacteria resistant to DOX and GEN were the least abundant in all treatments and were only found in the sandy loam soil (Table 2). For instance, GEN-resistant bacteria were already the lowest in USS (0.25%) and represented only 3.7% and 0.6% of corresponding THB counts in treatments SL80 and SL120, respectively. The same trend applies also for LEV-resistant bacteria in both soils despite being present in USS at 17% of total cultivable bacteria. However, the index of antibiotic resistance (ARB/THB) could be still considered significant for some antibiotics. In particular, high IAR values were calculated for AMX, AMP, AZI and to a less extent for ERY in soil S treated for 5 years with high to excessive sludge amounts of 80 and 120 t ha^−1^ year^−1^. Consequently, these indices were respectively about 80% for AMX, 60% for AMP and 76% and 66% for AZI (Table 2). The positive correlation between sludge dose and IAR was also observed for the same antibiotics in soil SL though at lower percentages.

The devastating flash flood that occurred 1 year after the fifth regular and presumably last sampling campaign resulted in disturbances of bacterial counts and distribution in both soil treatments. Total heterotrophic bacteria dropped significantly in all soil treatments but were still correlated to USS dose in both soils (*r* ˃ 0.9) (Table 2). For instance, THB in control treatments decreased by approximately 10 and 3 folds for soils S and SL, respectively. Nevertheless, the positive textural effect on bacterial counts was still obvious for soil SL compared to soil S. There were also disruptions in ARB counts in these two unamended soil treatments with respect to observations made 1 year earlier. In particular, AMX-, ERY-, LEV- and GEN-resistant bacteria disappeared completely in the sandy soil control. Bacterial counts and distribution in USS-amended soils experienced also variations with respect to the previous sampling campaign. In general, ARB numbers decreased in both soil treatments with a stronger impact observed in soil S. The latter showed a complete disappearance of bacteria resistant to AMX, AMP, DOX and LEV in all USS-treated plots. In contrast, GEN-resistant bacteria were detected in S120 treatment after the flash flood as compared to 2017 (Table 2). Sludge-treated plots of sandy loam texture were also affected but still showed resistant bacteria to all tested antibiotics. In any case, bacteria resistant to AZI and ERY were likely to be the less affected by the extreme climatic event of 2018.

### Multiple antibiotic susceptibility and resistance

The multiple resistance of the 52 morphologically selected isolates was investigated for 10 antibiotics as described in the “Multiple antibiotic resistance test” section. Resistant bacteria were found for all tested antibiotics (Table 3). Multi-antibiotic resistance of an isolate is defined as its ability to resist to at least three antibiotics, which means a MAR index ≥ 0.3 for the current investigation. Most of bacterial isolates (86.5%) were resistant to at least one antibiotic and fell into an index range of 0.1–0.2 as shown in Table 3. More precisely, 21 isolates were resistant to only one antibiotic (40.4%), 15 to two antibiotics (28.8%), 8 to three antibiotics (15.4%) and one isolate from USS to four antibiotics (1.9%). The seven remaining isolates (obtained mostly from unamended soils) showed sensitivity to all antibiotics (Table 3). Therefore, about 17% of morphologically isolated strains could be considered multi-antibiotic resistant. Besides, the presence of β-lactams (AMP, AMX, PEN) and macrolides (AZI, ERY) in exposure media induced the growth of the highest number of resistant bacteria. In fact, most of isolates were resistant to at least one of these two antibiotics families (61.5%). Among these, 36.5% of isolates were resistant to ERY, 32.7% to AZI and 17.3% to AMX or AMP or PEN (Table 3). Only one isolate (from USS) was resistant to fluoroquinolones (OFL and LEV).

**Table 3:** Antibiotic and metal resistance of 52 morphologically isolated strains from sludge and different soil treatments

Group	Multiple antibiotic resistance index (corresponding antibiotics)	Metal resistance	Number of tested isolates	% isolates	Origin of isolates
1	0	As / Be	7	13.5	4 (S0); 1 (S40); 2 (SL0)
2	0.1 (AZI)	As / Be	4	7.7	1 (S120); 1 (SL0); 2 (SL40)
3	0.1 (ERY)	As / Be / Cd	6	11.5	3 (S40); 3 (SL120)
4	0.1 (DOX)	As / Be	11	21.1	4 (SL40); 7 (SL80)
5	0.2 (AZI / ERY)	As / Be / Cd	13	25	2 (S80); 7 (S120); 4 (SL120)
6	0.2 ( CHL/GEN)	As / Be / Cd	1	1.9	1 (S120)
7	0.2 (LEV/OFL)	As / Be	1	1.9	1 (USS)
8	0.3 (AMP/AMX/PEN)	As / Be / Cd	8	15.4	5 (S120); 3 (SL80)
9	0.4 (AMP/AMX/PEN/CHL)	As / Be / Cd	1	1.9	1 (USS)
Total			52	100	

### Heavy metal resistance and species identification

This test was interpreted based on the absence/appearance of inhibition zones (˃ 1 mm) surrounding metal-impregnated discs. As shown in Table 3, all bacterial strains isolated from different THB and ARB plates were resistant to As and Be added at 20 mg L^−1^. Among these, 56% were also resistant to Cd, while the rest of strains (23) were inhibited by the same concentration in the exposure medium. In addition, Cd-resistant bacteria were only found in USS and USS-amended soils (Table 3). Out of the 52 isolates, 45 showed a dual resistance to at least one antibiotic and two metals. Sensitive strains (7) to all tested antibiotics showed resistance to only As and Be, while the nine confirmed multiple antibiotic-resistant isolates (MAR ≥ 0.3) were resistant to the three trace metals (Table 3).

### Species identification

**Fig. 1:**
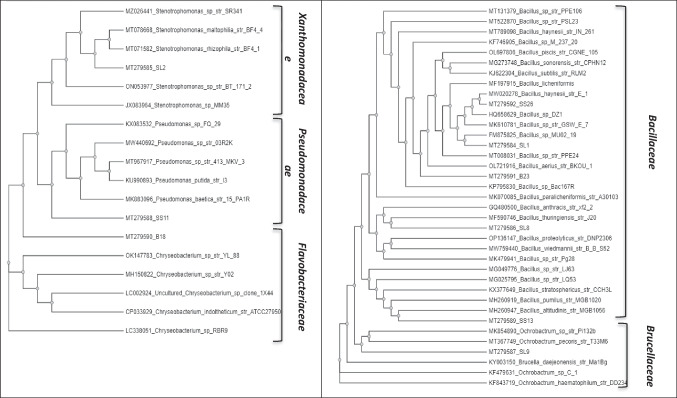
Phylogenetic tree of 16S rDNA gene sequences of selected strains resistant to tested antibiotics/metals and clustered according to corresponding bacterial families

Based on MAR results and subsequent metal resistance ability, the 52 isolates were divided in 9 groups as indicated in Table 3. One strain from each group was selected and identified by sequencing of 16S rDNA, which allowed the construction of a phylogenetic tree (Fig. 1). As shown in Table 4, the molecular identification showed that the selected strains belong to three phyla, namely, Firmicutes, Proteobacteria and Bacteroidetes with five representative genera: *Bacillus* (Bacillaceae), *Stenotrophomonas* (Xanthomonadaceae), *Ochrobactrum* (Brucellaceae), *Pseudomonas* (Pseudomonadaceae) and *Chryseobacterium* (Flavobacteriaceae). *Bacillus* was the dominant genus with five identified species out of the nine sequenced isolates. In this regard, soil-isolated *Bacillus licheniformis* DAS-1 was the sole selected species sensitive to all antibiotics. On the other hand, the two confirmed MAR and MRB species were *Bacillus* sp*.* MU02-19 and *Chryseobacterium indoltheticum* ATCC 27,950 isolated from soil and urban sewage sludge, respectively (Table 4). In addition to As, Be and Cd, *C. indoltheticum* was particularly resistant to all investigated β-lactams (PEN, AMX and AMP) and phenicols (CHL).

**Table 4:** Partial sequence analysis of 16 rDNA gene from selected isolates

Group	Isolate label	Accession number	Most related organisms	Phylogenetic group	Accession number*	Sequence similarity (%)	Sequence length (bp)	Analysed sample
1	Seq9: S26	MT279592	*Bacillus licheniformis strain DAS-1*	*Bacillaceae (Phylum: Firmicutes)*	KF664027	99	615	Soil
2	Seq4: SL9	MT279587	*Ochrobactrum pecoris strain 08RB2639*	*Brucellaceae* *(Phylum: Proteobacteria)*	NR_117053	99	550	Soil
3	Seq3: SL8	MT279586	*Bacillus thuringiensis strain J20*	*Bacillaceae (Phylum: Firmicutes)*	MF590746	97	458	Soil
4	Seq6: S13	MT279589	*Bacillus altitudinis* *strain MGB1056*	*Bacillaceae (Phylum: Firmicutes)*	MH260947	99	941	Soil
5	Seq2: SL2	MT279585	*Stenotrophomonas rhizophila strain BF4-1*	*Xanthomonadaceae* *(Phylum: Proteobacteria)*	MT071582	98	961	Soil
6	Seq5: S11	MT279588	*Pseudomonas baetica strain S15_PA1R*	*Pseudomonadaceae* *(Phylum: Proteobacteria)*	MK883096	99	923	Soil
7	Seq8: USS23	MT279591	*Bacillus* sp. *Bac167R*	*Bacillaceae (Phylum: Firmicutes)*	KP795830	99	875	Sewage sludge
8	Seq1: SL1	MT279584	*Bacillus* sp. *MU02-19*	*Bacillaceae* *(Phylum: Firmicutes)*	FM875825	97	179	Soil
9	Seq7: USS18	MT279590	*Chryseobacterium indoltheticum* *strain ATCC 27,950*	*Flavobacteriaceae* *(Phylum: Bacteroidetes)*	CP033929	98	847	Sewage sludge

^*^Accession number of the most related organism. When more than one sequence has the same similarity, only accession number of the first sequence is given

## Discussion

The agricultural valorization of urban sewage sludge as a biofertilizer brings not only organic carbon and nutrients to amended soils but also several compounds designated as stressors, of which are heavy metals, and recalcitrant natural or synthetic compounds, including antibiotic residues and/or antibiotic-resistant genes (Urra et al. 2019). Since bacteria have different degrees of tolerance or defensive responses against these harmful molecules, these latter can influence the composition of surviving communities in soil (Seiler and Berendonk 2012; Berendonk et al. 2015; Manaia et al.^ 2016^) and explain the variation of antibiotic and metal resistance with treatments.

### Antibiotic resistance in sludge and soil

Total heterotrophic bacteria in soils S and SL were significantly influenced by USS application and soil texture. In this regard, the significant dose-dependent increase of heterotrophs up to the highest counts observed in treatments S120 and SL120 has been previously reported for the same field study after the second, third and fourth USS application (Zoghlami et al. 2016; Hamdi et al. 2019; Hechmi et al. 2021). This improvement is related to the biostimulation and bioaugmentation effects of sludge-borne nutrients and microorganisms on amended soils (Hamdi et al. 2007; Wang et al. 2017; Balaganesh et al. 2020; Hechmi et al. 2020). In fact, the enhancement of soil fertility with the sludge dose has consistently been observed in both soils during previous sampling campaigns with significantly higher levels in soil SL (Zoghlami et al. 2016; Hamdi et al. 2019; Hechmi et al. 2020, 2021). Consequently, the influence of soil texture on THB counts is obvious as higher clay fraction, SOM and CEC in the sandy loam soil result in a greater water and nutrient adsorption capacity with respect to the coarser soil S (Ward and Oades 1993). Moreover, higher bacterial populations in finer soil particles (< 2 μm) have also been attributed to the role of the clay layered structure in protecting them against desiccation, radiation and predation (Belnap 2003; Mohammad 2015).

Various studies have shown that drugs including antibiotics are not completely metabolized in human and animal bodies after administration and could be excreted either as parent compounds or as metabolites via urine and faeces (Zhou et al. 2021). They may enter the environment directly along with excreta (e.g. through fresh dung deposition or land application of manure) or via the discharge, landfilling or reuse of raw/treated effluents and urban sewage sludge (Gao et al. 2012; McEneff et al. 2014). The latter represents a reservoir of ARB and ARGs and contains compounds that affect bacterial communities and contribute to their multiplication and dissemination in soil after amendments (Di Cesare et al. 2016; Marano and Cytryn 2017; Urra et al. 2019). In this study, the used USS contained resistant bacteria to all tested antibiotics (Table 2). This proves that these most prescribed antibiotics in Tunisia do exist in human excreta and induce bacterial resistance in USS as a wastewater treatment by-product. In one of the few studies on antibiotic residues in Tunisian urban wastewater, Harrabi et al. (2018) quantified 11 antibiotics in raw and treated effluents based on available calibration standards. Among them, enrofloxacin (a veterinary drug) was the most abundant in raw wastewater (400.2 ng L^−1^), while AMP and AZI had concentrations of 75.4 and 135.45 ng L^−1^, respectively. Surprisingly, they did not detect the most prescribed PEN and AMX in raw effluents probably because of their chemical instability and easy transformation through hydrolysis (Knapp et al. 2011). In another investigation carried out in four wastewater treatment plants (WWTPs) in Grand Tunis (capital city and suburbs), Tahrani et al. (2017) identified 34 antibiotics among which 33 were different from those addressed in the current study. Consequently, they reported concentrations of ERY varying from zero to excessive concentrations of 16.4 ng L^−1^ in raw wastewater depending on the sampling site and season. In any case, these limited available data from the current region of study emphasize on the overconsumption and high environmental concern of antibiotics in terms of ubiquity and variability.

The indices of antibiotic resistance in USS varied between lowest values for GEN (0.25%) to highest values for AMX (18%), DOX (24%) and AMP and AZI (38%). Despite the fact that Tunisia is among antibiotic overusers worldwide (Klein et al. 2018), details on the most prescribed ones are insufficient. Mansour (2018) reported that antibiotics use increased by 38% between 2005 and 2013 and is still increasing with a preponderance of β-lactams such as AMP and AMX. This may explain the presence of corresponding resistant bacteria in USS at significant levels. In contrast, Kallel et al. (2005) conducted a survey on antibiotics consumption in a Tunisian university hospital and found that GEN was the most prescribed antibiotic against infections. This indicates that the actual counts of ARB in the sludge sample were largely influenced by the period and site of study, the origin of collected effluents and the fate of antibiotics in relation with wastewater treatment technologies and USS storage conditions at WWTPs (Harrabi et al. 2018). In any case, recent local reports indicate that the excessive use of antibiotics and subsequent microbial resistance have been further aggravated since 2020 with COVID-19 infections being mostly treated using cefotaxime, ofloxacin and azithromycin (Ben Saida et al. 2020; Ghali et al. 2021).

On the other hand, several parameters are involved in the emergence and environmental spread of antibiotic-resistant bacteria (Martínez and Baquero 2014). Accordingly, both unamended soils already contained colonies resistant to AMX and LEV (S0) and AZI and ERY (S0 and SL0) as indicated in Table 2. It has been shown that non-exposure to antibiotics can also lead to antibiotic resistance selection, accumulation and dissemination through mutagenicity and horizontal gene transfer (Andersson and Hughes 2010; Diard et al. 2017; Iwu et al. 2020). Factors such as ARGs, heavy metals, biocides (Singer et al. 2016), solvents (Fernandes et al. 2003) and plant-derived chemicals (Friedman 2015) have all been identified as antibiotic resistance‐conferring precursors. Since both experimental plots (S and SL) belong to a research station that has been operating for decades, it was expected that they had been used in various research studies before the implementation of the current project in 2012. According to the station’s personnel, previous experiments had been principally cropping trials using treated wastewater and various conventional and non-conventional agricultural inputs. All these activities might have induced intrinsic antibiotic resistance in both experimental plots prior to current investigation.

As mentioned previously, sewage sludge represents a reservoir of ARB and contains residual antibiotics and other compounds that may contribute to antibiotic resistance acquisition, multiplication and dissemination in sludge-amended soils (Bondarczuk et al. 2016). This was confirmed by the appearance of resistant bacteria to the seven selected antibiotics in treated soils after five annual applications (Table 2). Several studies have shown that raw or treated wastewater, sewage sludge and animal excreta are important routes of transmission of ARB, ARGs and residual antibiotics to the soil environment (Li 2014; Massé et al. 2014; Bondarczuk et al. 2016; Carvalho and Santos 2016). Chen et al. (2018) pointed out that once these inputs are used in agricultural systems, they would exert discerning pressure on indigenous microbial communities as well as augment the ARB and ARGs presence in the soil. Since bacteria have different degrees of tolerance or defensive responses against loaded molecules or stressors, these latter can influence the composition of surviving communities in soil and explain the variation of ARB numbers with soil treatments (Seiler and Berendonk 2012; Berendonk et al. 2015; Manaia et al. 2016). This was eventually observed in the current study as ARB counts varied largely with antibiotics type. On one hand, the general positive correlations observed between the sludge dose and ARB abundance in both soils could be mainly explained by a long-term bioaugmentation effect through the dose-dependent addition of bacterial resistome, for instance, AZI-resistant bacteria augmented by about 70- to 80-fold in S120 and SL120 treatments, respectively, compared to unamended controls. Chen et al. (2016) noticed also a similar dose-dependent increase of ARG diversity and detection frequency in soils treated on the long term with sludge doses up to 36 t ha^−1^. On the other hand, the highest numbers of ARB in all treatments were observed for AMP and AMX in S120 and SL120, respectively, which corroborates the observations made by Mansour (2018) on the dominance of β-lactams use in Tunisia between 2005 and 2013. This was also proved by the important IAR percentages of both antibiotics especially in treatment S120 (˃ 60%). Rahubi et al. (2014) registered 79.2% of resistant coliforms to AMP in soil amended with 28.6 t ha^−1^ of sewage sludge against 46.4% in untreated control. Resistance to the macrolide AZI showed also significant enhancement in both soils driven principally by a high content of AZI-resistant bacteria in the used sludge (332 × 10^5^ CFU g^−1^). This issue is of particular concern as recent studies have reported on a worldwide overuse of AZI to treat COVID-19 (Ben Saida et al. 2020; Adam et al. 2021; Echeverría-Esnal et al. 2021).

Soil texture is a dominant abiotic parameter that controls microbial activity by influencing pore size distribution, water and nutrient availability and specific surface area (Scott et al. 1996). Accordingly, several research studies have highlighted the role of soil type/texture in controlling the fate of ARB and ARGs in soil after incorporation of various organic materials (Zhang et al. 2018; Guron et al. 2019; Macedo et al. 2020). In contrast to current and previous outcomes of THB variation between soils S and SL, the influence of texture on ARB distribution remains ambiguous in this study. Consequently, the positive role of fine soil particles in providing favourable growth conditions for culturable bacteria was not evident for ARB in soil SL treatments compared to soil S treatments (Hamdi et al. 2019; Hechmi et al. 2020). On the contrary, ERY- and AZI-resistant bacteria were more numerous in soil S resulting in higher IAR percentages as shown in Table 2. Blau et al. (2018) found the effects of antibiotic-spiked manure on relative ARGs abundances and soil bacterial community composition strikingly more pronounced in sandy soil than in loamy soil. It is possible that the specific factors controlling the textural effect on total heterotrophs under the described experimental conditions do not apply to antibiotic-resistant bacteria.

The type of antibiotics was likely to play a role in the distribution of ARB as well. In particular, LEV- and DOX-resistant bacteria were present at high counts in USS but were significantly low or absent in most soil treatments after five consecutive sludge applications. Burch et al. (2014) reported the decline of five erythromycin and tetracycline resistance genes and the integrase of class 1 integrons (*intI1*) in two USS-amended soils (sandy and silt-loam) after incubation in microcosms. It is likely that these two particular ARB were more vulnerable to the prevailing soil conditions of this field study than the rest of resistant strains. For instance, Pérez-Valera et al. (2019) highlighted the role of native soil microorganisms in hindering the enrichment of topsoil with tetracycline antibiotic resistance genes (TET-r) following manure application by preventing the establishment of their bacterial hosts. Comparable results were also found for mixtures of soil with manure or biogas digestate in microcosms suggesting that increased competition for nutrients, antibiosis or smaller niche availability prevent the establishment of antibiotic-resistant or pathogenic bacteria from the applied organic fertilizers (Chen et al. 2017).

The extreme flash flood of September 2018 affected negatively both THB and ARB communities in terms of numbers and distribution compared to observations made 1 year earlier. The torrential rain resulted in an intense surface runoff and water stagnation in S and SL plots that lasted for several days despite their light texture. In their simulated flood experiments, Unger et al. (2009) found that a 5-week stagnant flood conditions decreased the microbial biomass at field scale. It has been reported that an increase in soil moisture leads to a decrease in the redox potential through rapid consumption of oxygen by microorganisms, which creates reducing anaerobic conditions (Tokarz and Urban 2015). Accordingly, Song et al. (2008) investigated the diversity of soil microorganisms associated with changes in the oxidation–reduction potential and found that the highest abundance (16% of the abundance of all soil microorganisms) in anaerobic conditions was shown by Gram-positive bacteria, which reflects their low sensitivity to oxygen deficiency compared with other microbial groups. In this study, the general decrease of bacterial counts in all soil treatments indicates that part of THB (including ARB) were sensitive to the sudden disturbance of soil conditions as immediate shifts in microbial community structure and counts are expected when anaerobic conditions develop from flooding (Unger et al. 2009). In addition, it has been shown that water movements in soil including runoff, leaching and infiltration affect the ability of microorganisms to attach/detach from different binding sites of the solid phase (Pachepsky et al. 2008; Critzer and Doyle 2010). Consequently, the observed decrease of THB/ARB counts in the 0–20 cm profile may also be the consequence of a certain “washout” of less competitive bacterial strains over sorption sites. Several morphological characteristics and genes are correlated with the affinity of bacteria to become attached to mineral surfaces, and the genetic variation may even cause differences in the attachment of several bacteria strains to the same soil particle (Celico et al. 2004; Pachepsky et al. 2008). On the other hand, it is possible that THB and ARB communities were also affected by a natural decline because the last USS amendment occurred in fall 2016. This means that 2 years had already elapsed between the final sludge application and the sampling of 2018. Consequently, the termination of USS-related enrichment with ARB, ARGs, residual antibiotics and nutrients from one side and the possible biodegradation of remaining antibiotics in soil from the other side might have all participated in this decline as well (Gavalchin and Katz 1994; Blau et al. 2018).

### Co-occurrence of antibiotic and metal resistance

The multiple resistance of 52 morphologically isolated colonies from different THB and ARB plates was tested against 10 antibiotics and 3 trace metals. Out of these 52 bacterial strains, seven isolates from THB plates were sensitive to all tested antibiotics. Since total heterotrophs represent all cultivable bacteria grown on non-selective growth media, it is anticipated that they will have a broad spectrum of responses to any environmental stressor (0 to 100% inhibition) (Zhang et al. 2015). A representative strain selected from this group of antibiotic-susceptible isolates was identified as *Bacillus licheniformis* DAS-1, which is a Gram-positive, spore-forming bacterium species widely distributed as a saprophytic organism in the soil environment. *B. licheniformis* is pathogenic to immunocompromised patients but is also an important facultative anaerobe with numerous commercial, industrial and agricultural applications (Salkinoja-Salonen et al. 1999; Rey et al. 2004). Banoon et al. (2020) tested the resistance profile of 56 soil *B. licheniformis* isolates (not specified) against 27 antibiotics including similar compounds to the current studies. They found also 100% sensitivity to GEN and 10 other antibiotics, while 3%, 23% and 93% were resistant to CHL, AMX and AMP, respectively. In their study on putative antibiotic resistance genes present in 73 *B. licheniformis* strains, Agersø et al. (2019) identified an intrinsic CHL resistance gene (cat), qualified as part of the ancient resistome in all strains. This was not actually the case in the current study, but the fact that DAS-1 strain might show resistance to other antibiotics than the 10 tested ones could be also conceivable (Agersø et al. 2019; Banoon et al. 2020). To our best of knowledge, no research studies have addressed antibiotic resistance of the specific *B. licheniformis* DAS-1 strain. However, Tripti et al. (2014) put into evidence its potential uptake and reduction of different arsenic forms under different stresses. Accordingly, the resistance of DAS-1 strain to As added at 20 mg L^−1^ was confirmed in this study (Table 3).

Metal resistance is a common phenotype in many microorganisms that are exposed to metals in their habitats. Several studies have indicated that metallic elements could be also an important driver to select antibiotic resistance (Pal et al. 2017; Chen et al. 2019a, b; Dickinson et al. 2019). Sun et al. (2021) observed synergistic relationships between the majority of studied MRGs and ARGs in activated sludge. Chen et al. (2015) found that exposure to extremely low concentrations of zinc, copper and arsenate induced the resistance of bacterium LSJC7 towards tetracycline. They suggested that such acquired antibiotic resistance might be ubiquitous among various microbial species, which plays a role in the emergence and spread of antibiotic resistance in metal and antibiotic co-contaminated environments. Evidences for co-selection of ARGs and MRGs (metal resistance genes) have been reported in a variety of environments over the past several decades (Li et al. 2017). More precisely, retrospective studies showed that metal and antibiotic resistances co-occurred in isolates from early in the age of wide-scale antibiotic use (Hobman and Crossman 2015). Thus, the co-selection of bacterial strains or mobile genetic elements that they carry is mainly caused by co- or cross-resistance mechanisms (Pal et al. 2017). The co-resistance occurs when two resistance genes are physically co-located in the same elements of a cell, such as a chromosome, a plasmid or a transposon (Sun et al. 2021). In contrast, cross-resistance occurs when a single mechanism (efflux pump) provides resistance to different compounds simultaneously (Chapman 2003; Pal et al. 2017).

In the present study, the occurrence of dual resistance in the 52 isolates was tested in presence of Cd, As and Be. These non-essential metals are used in various industries and can be identified in a variety of environmental media such as the air, surface water, ground water, wastewater, leachate, sediments and soil (Su et al. 2014; Shah et al. 2016). While the toxicities of As and Cd have long been categorized (Clemens and Ma 2016; Balali-Mood et al. 2021), the least addressed Be could be also a bioaccessible pollutant in soil ecosystems (Rashidul Islam et al. 2022). According to Shah et al. (2016), it may severely affect the performance of crops and/or transfer to humans via the food chain. Because Be^2+^ is a highly charged small ion, it can easily get into many tissues and cells of the host, where it specifically targets cell nuclei, inhibiting many enzymes, including those used for DNA synthesis. The toxicity of Be is exacerbated by the fact that the human body has no means to control its levels (Gobato and Heidari 2018). In this investigation, all morphologically isolated strains from different THB and ARB plates showed resistance to As and Be independently of antibiotic type or MAR index. To our best of knowledge, this is the first study investigating the occurrence of dual antibiotic/beryllium resistance in urban sludge and soil samples. Besides, exposure to Cd at 20 mg L^−1^ resulted in only 56% of resistant isolates indicating higher toxic effects than As or Be under the described experimental conditions. Increases of Cd concentrations have been generally linked to reduction in microbial populations and disturbances in respirometric and enzyme activities (Vig et al. 2003; Xiao et al. 2020). Depending on bacterial strains, previous studies reported various responses to Cd exposure ranging from total resistance for excessive concentrations (Fritz et al. 2000) to inhibitory effects at much lower levels (Dar 1996). Besides, the intrinsic concentrations of Cd, Be and As in both soils and in USS particularly (4, 10 and 17 mg kg^−1^) were likely to induce a proportional degree of resistance (or adaptation) in culturable bacteria. This was expressed by stronger sensitivity to Cd when the three trace metals were added at high bioavailable levels of 20 mg L^−1^ each (Bruins et al. 2000).

Among all selected isolates, only one *Bacillus* sp. Bac167R strain isolated from USS showed resistant to the two tested fluoroquinolones, namely, OFL (2nd generation) and LEV (3rd generation) (Tables 3 and 4). The quinolones are a novel synthetic class of antimicrobial agents classified into generations based on their antibacterial spectra (Lilley et al. 2000). Nearly all quinolone antibiotics in use are fluoroquinolones, which contain a fluorine atom in their chemical structure and are effective against both Gram-negative and Gram-positive bacteria (Heeb et al. 2011). Among 13 antibiotics detected in raw wastewater of Sfax city (Tunisia), Harrabi et al. (2018) noted the dominance of fluoroquinolones with highest concentrations for enrofloxacin (400.2 ng L^−1^), followed by ciprofloxacin (330.33 ng L^−1^) and ofloxacin (175.01 ng L^−1^). Although several *Bacillus* species have shown resistance to fluoroquinolones in various studies on sludge (Lee et al. 2017; Huang et al. 2020), the specific resistance of *Bacillus* sp. Bac167R strain has never been previously reported.

Significant multi-antibiotic resistance (MAR ≥ 0.3) was observed in nine morphologically isolated strains: eight isolates to PEN, AMP and AMX; and one isolate to PEN, AMX, AMP and CHL. All these MAR strains were also concomitantly resistant to Cd, As and Be as shown in Table 3. Therefore, they could be considered of potential health concern to humans among the 52 strains isolated from soil and urban sewage sludge. Li et al. (2017) confirmed that the signatures of antibiotic and metal resistance co-occurrence are much more frequent and the distance linkages between ARGs and MRGs are much more intimate in human pathogens than those less human-associated bacteria. The common characteristic of these nine isolates is their multiple resistance to β-lactam antibiotics (AMP, AMX and PEN). Beta-lactams are one of the most commonly prescribed antibiotic classes with numerous clinical indications since their advent starting from the 1930s. It has been estimated that the annual expenditure for these antibiotics amounts to approx. 15 billion of USD and makes up 65% of the total antibiotics market (Thakuria and Lahon 2013). In the current region of study, one inventory report on antibiotic consumption by type revealed also the dominance of β-lactams use between 2005 and 2013 (Mansour 2018). Consequently, their overuse clashes with the worrying phenomenon of the development and environmental spread of β-lactam resistance, which represents a global health issue. This has been worsened by the outbreak of COVID-19 pandemic and subsequent excessive administration of various antibiotics to patients including β-lactams (Novy et al. 2020). In this regard, wastewater treatment operations and subsequent output products have been shown to constitute the major pathways for MAR acquisition and environmental dispersion (Rizzo et al. 2013; Jardine et al. 2019).

*Chryseobacterium indoltheticum* (ATCC 27,950) was the most multiple resistant strain among all selected isolates and originated from urban sewage sludge. In addition to its resistance to the three broad-spectrum β-lactams, it showed also resistance to CHL. The latter was the first mass-produced antibiotic and has been primarily used as an eye ointment or eye drops to treat human ocular infections such as conjunctivitis and blepharitis (Kmietovicz 2021). Chloramphenicol could be exceptionally used by mouth or injection when safer antibiotics cannot be found but has been banned in food animal production due to its potential toxicity risks to humans (Hanekamp and Bast 2015). A study conducted in Tunisia by Freitas et al. (2017) on phenicol resistance in wastewater indicated that corresponding *fexA* and *optrA* resistance genes co-occurred in two *Enterococcus faecalis* isolates, testifying to the presence of CHL in excreta. Accordingly, Tahrani et al. (2016) confirmed the existence of CHL in effluents of two Tunisian WWTPs (3.3 and 0.5 μg L^−1^, respectively) as well as in seawater samples collected close to fish farming sites at levels up to 15.6 μg L^−1^. This indicates on its potential use in aquaculture activities of local companies off the Mediterranean coast and possible transfer to consumers via CHL-treated fish as well (Chelossi et al. 2003).

*Chryseobacterium* species are inherently resistant to a wide spectrum of heavy metals and antibiotics, including tetracyclines, erythromycin, linezolid, polymyxins, aminoglycosides, chloramphenicol and several β-lactams (Benmalek et al. 2014; Izaguirre-Anariba and Sivapalan 2020). Most of published studies have particularly addressed the pathogenicity and resistance of *Chryseobacterium indologenes* strains, which are aerobic, Gram negative, nonfermentative rods that are intrinsically multidrug resistant (Lin et al. 2010; Izaguirre-Anariba and Sivapalan 2020). Reported infections include bacteremia, pneumonia, meningitis, myositis, keratitis and indwelling devices (Izaguirre-Anariba and Sivapalan 2020). For instance, *C. indologenes* (B18) is an emerging pathogen, which poses a threat in clinical healthcare setting due to its multidrug-resistant phenotype and its common association with nosocomial infections (Yu et al. 2016). Published research studies on *Chryseobacterium indoltheticum* pathogenicity and antibiotic resistance are extremely scarce. Tsôeu et al. (2016) reported that among 14 *Chryseobacterium* species isolated from dairy products, two C. *indoltheticum* isolates were identified and showed a certain potential to cause spoilage defects in dairy products because they were able to utilize a wide range of compounds in the BIOLOG system. In their investigation on multidrug resistance in *Chryseobacterium*, Michel et al. (2005) reported that most *Chryseobacterium* sp. isolates of aquatic origin and reference strains *C. indoltheticum* (LMG 4025^ T^ and 13,342) exhibited considerable multi-resistance to most antimicrobial drug families including chloramphenicol and florfenicol. The current study is probably the first of its kind to report the multiple antibiotic and metal resistance of the specific strain *C. indoltheticum* (ATCC 27,950) isolated from urban sewage sludge destined to agricultural land application.

## Conclusions

This study revealed that repeated land applications of urban sewage sludge at increasing rates could influence the abundance and co-occurrence of antibiotic- and metal-resistant bacteria with respect to unamended soils. This was further highlighted by carrying out the field trial in total absence of plants, which might mask intrinsic soil-sludge interactions and microbial abundances and/or behaviour. As a complex biowaste of dominant human origin, urban sewage sludge may contain residual antibiotics and corresponding resistant bacteria and/or genes especially in countries where antibiotic overconsumption and self-medication are prevailing. This issue has been globally accentuated by the COVID-19 pandemic and the ubiquitous prescription of antibiotics to infected persons. While the effect of soil texture on ARB distribution was not as obvious as for THB or physico-chemical properties, an extreme flash flood resulted in a general attenuation of ARB counts that was further magnified by the termination of sludge amendments. Outcomes showed also that nine morphologically selected isolates had significant multiple antibiotic resistance including one identified sludge-borne strain (*Chryseobacterium indoltheticum* ATCC 27,950) concurrently resistant to four antibiotics and three toxic metals. Therefore, potential threats to human health through biologically contaminated crops grown on sludge-amended soils should be anticipated in case of severe ARB contamination. Further investigations are currently directed at deeply identifying antibiotic resistome, classes and residual concentrations in USS and different soil treatments.

## Data Availability

All data generated or analysed during this study are included in this published article.
